# Magnetic Noise Prediction and Evaluation in Tunneling Magnetoresistance Sensors

**DOI:** 10.3390/s18093055

**Published:** 2018-09-12

**Authors:** Jakub Chęciński, Piotr Wiśniowski, Marek Frankowski, Tomasz Stobiecki

**Affiliations:** 1 Department of Electronics, AGH University of Science and Technology, Al. Mickiewicza 30, 30-059 Kraków, Poland; piotr.wisniowski@agh.edu.pl (P.W.); mfrankow@agh.edu.pl (M.F.); stobieck@agh.edu.pl (T.S.); 2 Faculty of Physics and Applied Computer Science, AGH University of Science and Technology, Al. Mickiewicza 30, 30-059 Kraków, Poland

**Keywords:** magnetic field sensing, tunneling magnetoresistance devices, noise modeling, 1/f noise, white noise

## Abstract

We propose a simple model for prediction of magnetic noise level in tunneling magnetoresistance (TMR) sensors. The model reproduces experimental magnetic 1/f and white noise components, which are dependent on sensors resistance and field sensitivity. The exact character of this dependence is determined by comparing the results with experimental data using a statistical cross-validation procedure. We show that the model is able to correctly predict magnetic noise level for systems within wide range of resistance, volume and sensitivity, and that it can be used as a robust method for noise evaluation in TMR sensors based on a small number of easily measurable parameters only.

## 1. Introduction

Magnetic field detection by high sensitivity sensors based on tunneling magnetoresistive (TMR) devices has great potential for applications where high sensitivity, wide dynamic range, high spatial resolution and low power consumption are important. These sensors can be used in low and ultra-low magnetic field detection (biosensing and compass), high performance rotation and angle measurements (motor shaft, steering wheel, and anti-lock braking system) and current sensing (over current protection and high speed current monitoring) [1,2,3,4]. In most of these applications, the field detection, which is determined by the field sensitivity and noise level of the sensors, is the critical parameter that determines a sensor performance.

In principle, the sensors present both electronic and magnetic noise. It has been shown (see, e.g., [5,6]), however, that the magnetic noise dominates the electronic noise in high sensitivity TMR sensors. The magnetic noise appears during the sensing layer magnetization rotation between the saturated states of a resistance vs. magnetic field (*R*-*H*) curve (i.e., a transfer curve) and contains frequency dependent (1/f) and frequency independent (white) components. Both noise components depend on bias conditions as well as on several specific TMR sensors materials and design parameters [7,8,9], such as sensing layer volume, saturation magnetization, anisotropy field, Gilbert damping and susceptibility.

However, for predicting TMR sensors magnetic field detection performance, it is of great importance to have a few easily measurable sensor parameters that could be used to estimate the noise level of the sensors. Indeed, it has been shown that the magnetic noise of TMR sensors strongly correlates with derivative of a transfer curve (*dR*/*dH*) [5,10,11]. Therefore, we can define field sensitivity (*FS*) as a product of sensors bias current (*I_b_*) and the derivative, *FS* = *I_b_ dR*/*dH*. Because *dR*/*dH* depends on the resistance of the sensor, both the resistance and field sensitivity can be used to predict and evaluate the sensor’s noise and therefore the field detection.

To predict magnetic noise level of the TMR sensors, we developed a simple model that generates 1/f and white noise components. These components are related to sensor resistance and field sensitivity by polynomial dependencies. We show that the model sufficiently accurately reproduces experimental noise. Thus, it can be used for effective and robust prediction and evaluation of magnetic noise in TMR sensors with very different (three orders of magnitude range) values of resistance and field sensitivity.

## 2. Methods

**Figure 1:**
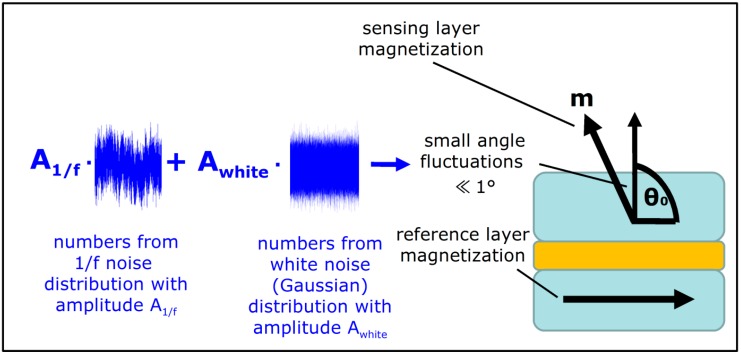
Illustration of the magnetic noise model. Both 1/f noise and white noise contribute to the small angle fluctuations of the sensing layer magnetization.

To reproduce magnetic noise of TMR sensors, we introduce fluctuation of magnetization angle θ of the sensing layer with respect to the reference layer. The angle fluctuates in time, and the fluctuation behavior is controlled by two independent stochastic processes, one that produces frequency dependent noise (1/f-like) and another that produces frequency independent noise (white noise) (see Figure 1). The amplitudes of these two processes, *A*_1/*f*_ and *A_white_*, respectively, are the numerical parameters of the model, which will control the noise generation. To provide a quantitative prediction of noise levels, these parameters are tied to easily measurable sensor parameters such as resistance and field sensitivity, as described in Section 3.

**Figure 2:**
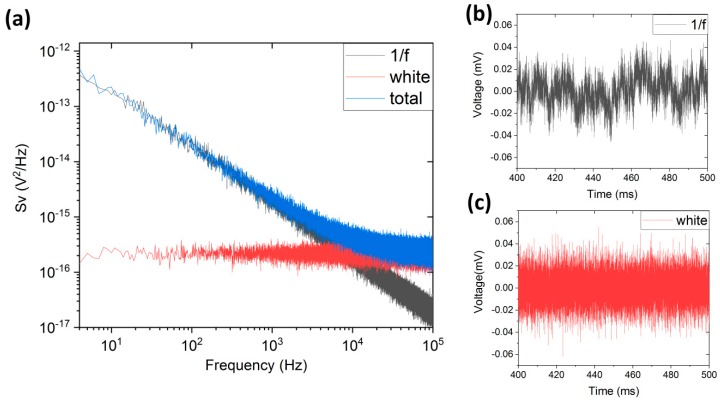
An example frequency-domain output (**a**) generated by 1/f-like stochastic process (**b**) and white noise-like stochastic process (**c**).

By describing the sensing layer of a TMR sensor with a single angle quantity, we can obtain a time series that corresponds to fluctuating magnetization. We used a standard formula for tunnel magnetoresistance of TMR sensors:

(1) $$R\left(\theta \right)=\frac{R_{AP}+R_{P}}{2}-\frac{R_{AP}-R_{P}}{2}\cdot \cos\theta \equiv R_{0}-∆R\cdot \cos\theta ,$$

where resistance *R* depends on the angle θ between the reference and the sensing layer magnetization and *R_AP_* and *R_P_* are resistances of anti-parallel and parallel state, respectively. Since we were interested in sensors operating in the highest sensitivity point on the transfer curve, θ was close to 90° in our case, with the deviations from 90° originating from fluctuating magnetization generated by the stochastic processes:

(2) $$\theta \left(t\right)=\theta_{0}+\delta \theta \left(t\right)\approx 90{}^{\circ}+\delta \theta \left(t\right),$$

(3) $$\delta \theta \left(t\right)=A_{1/f}\varepsilon_{1/f}\left(t\right)+A_{white}\varepsilon_{white}\left(t\right),$$

where $\varepsilon_{1/f}$ and $\varepsilon_{white}$ represent the stochastic processes generating normalized noise with unit variance of 1/f and white characteristics, respectively. Once the resistance time series was obtained, we multiplied the result by the bias current to obtain voltage as a function of time *V*(*t*). We performed Fast Fourier Transform on *V*(*t*) to receive a frequency-domain noise output. An example result, averaged 50 times, is shown in Figure 2a for 1/f-like stochastic process (black), white noise-like stochastic process (red) and both processes acting simultaneously (blue). For reference, the figure also includes example *V*(*t*) curves in the presence of 1/f-like (Figure 2b) and white noise-like (Figure 2c) stochastic process.

To produce a noise characteristic with our noise model, we need to provide sensors and stochastic process parameters and bias conditions. Thus, we need to know the values of *R*_0_ and Δ*R* (or, equivalently, *R_P_* and *R_AP_*) as defined in Equation (1), the bias current *I_b_* (or, equivalently, bias voltage *V_b_*) and to specify the values of two amplitudes *A*_1/*f*_ and *A_white_* connected with the stochastic processes. In the next section, we describe an algorithm to determine the relationship between *A*_1/*f*_ and *A_white_* and the experimental quantities *R*_0_, Δ*R*, *V_b_*, *FS*, and *Ω* (where *V_b_* is the bias voltage, equal to the product of *R*_0_ and *I_b_*, and *Ω* is the sensing layer volume). We show that these quantities, which are commonly used in sensor technology, can provide full information needed to predict the noise characteristics for a given sensor.

## 3. Results

### 3.1. Sensors Noise

**Figure 3:**
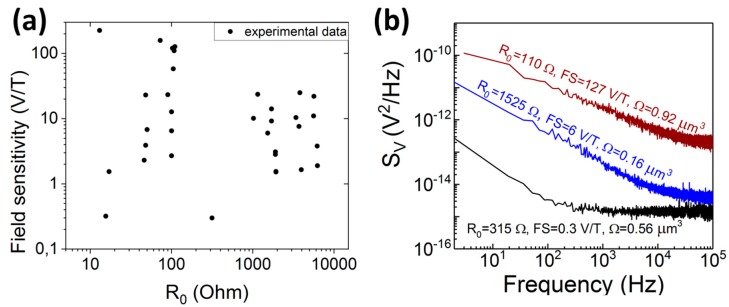
Resistance and field sensitivity of TMR sensors (**a**). Noise characteristics of selected sensors with different resistance, volume and field sensitivity values (**b**).

To determine the relationship between *A*_1/*f*_ and *A_white_* and the sensor parameters such as resistance and field sensitivity, we measured noise for TMR sensors with wide resistance range (from 13 Ω to 6250 Ω), volume of the free layer (0.029 μm^3^ to 13 μm^3^) and field sensitivity (from 0.3 V/T to 225 V/T) (see Figure 3a). Further details about the sensors can be found in [12,13]. The sensors’ noise (Figure 3b) was measured in a shielded doubled wall box, containing battery-powered low-noise amplifiers (voltage noise 4.7 nV/Hz^0.5^ and 6.4 nV/Hz^0.5^), voltage bias circuit, and a pair of coils generating a bias magnetic field. The noise power spectral density was recorded by the spectrum analyzer technique.

**Figure 4:**
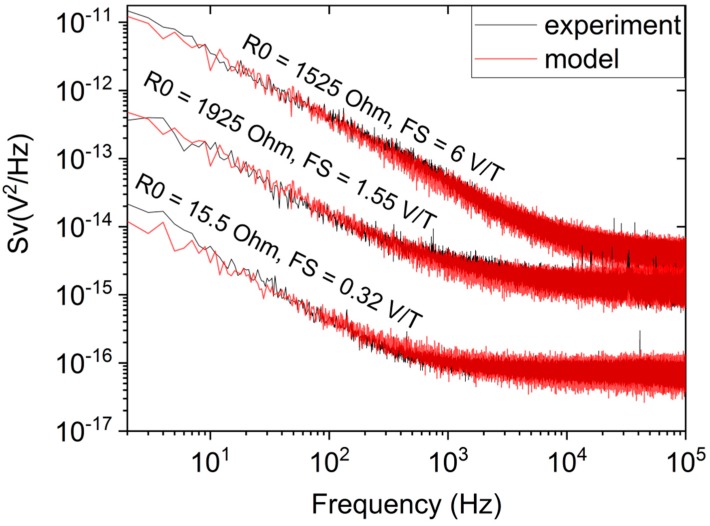
Comparison between experimental noise characteristics (solid black line) and their corresponding best matches in the model (transparent red line) obtained for three representative sensors.

For each measured sensor noise characteristic, we performed several thousand simulations based on our model with different pairs of *A*_1/*f*_ and *A_white_* values to find the pair that would produce the best match with the measured noise. An example result can be seen in Figure 4, where we show three experimental characteristics superimposed onto their best fitting matches in the model. The final *A*_1/*f*_ and *A_white_* parameters, determined for all investigated samples and normalized to 1 μm^3^ sensing layer volume, are listed in Appendix A. The next step is to identify the dependence between the obtained *A*_1/*f*_, *A_white_* and selected experimental parameters.

### 3.2. Noise Prediction and Evaluation

To predict the noise levels of TMR sensors using our model, we performed a statistical analysis scheme. The scheme was based on cross-validation technique, which is commonly utilized in statistics and data science [14,15]. Since we already accounted for the sensing layer volume *Ω* by normalizing the noise coefficients, only four possible independent predictors—*R*_0_, Δ*R*, *V_b_* and *FS*—remained. However, we found that the correlation between *R*_0_ and Δ*R* was very strong (correlation coefficient *ρ*_*R*_0___,Δ*R*_ > 0.98), so that models containing both variables could be statistically unreliable. As a result, we decided to drop Δ*R* from the possible predictors list and use *R*_0_ only. We note here that *R*_0_ displayed stronger correlations with both target variables, *A*_1/*f*_ and *A_white_*, than Δ*R* (−0.67 and −0.82 vs. −0.59 and −0.77, respectively), making it a better candidate for a predictor.

The available data points (*R*_0_, *Vb*, and *FS*) were randomly divided into N = 5 different sets of equal size. During each of N steps of the employed algorithm, a different one of those sets was treated as a validation sample, whereas all the others were treated as a training sample for a model candidate. Since the candidate model was fitted using only the training set part of the data, the comparison between its prediction and the actual noise level registered in the validation set part of the data provides a good estimate of the prediction error. By minimizing this prediction error, we can avoid overfitting and obtain a model that will be able to estimate noise levels of TMR sensors. Because of vast differences among sensor parameter levels (multiple orders of magnitude), we decided to perform the cross-validation scheme based on logarithmized quantities, as depicted in Table A1 in Appendix A.

**Figure 5:**
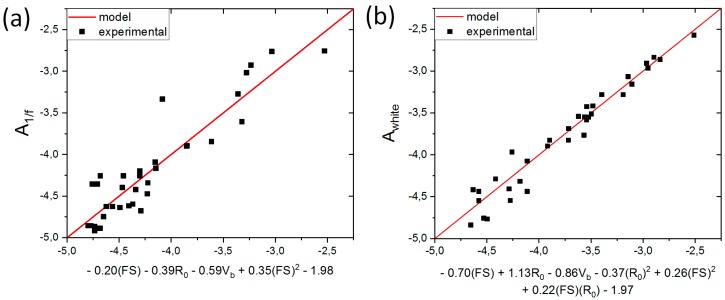
Illustration of the model prediction found for: 1/f-type noise (**a**); and white noise (**b**). In both charts, the respective noise coefficient is shown as a function of the specific expression identified, for both the regression model (red line) and the experimental data (black squares). All variables presented in this chart are logarithmized.

We tested different regression models, using cross-validation error as the criterion for comparing them to each other. We considered the following set of possible predictor variables: {*R*_0_, *FS*, *V_b_*, (*R*_0_)^2^, (*FS*)^2^, (*V_b_*)^2^, (*R*_0_)^3^, (*FS*)^3^, (*V_b_*)^3^, *R*_0_*·FS*, *R*_0_*·V_b_*, *FS·V_b_*, (*R*_0_)^2^*·FS*, (*R*_0_)^2^*·V_b_*, *R*_0_*·*(*FS*)^2^, *R*_0_*·*(*V_b_*)^2^, (*FS*)^2^*·V_b_*, *FS·*(*V_b_*)^2^}. Each model was constructed by selecting a subset of variables from this set and fitting the resultant expression to the available data using the previously described approach. Because of the large amount of possible combinations, we utilized the forward stepwise regression scheme [14,15] for both *A*_1/*f*_ and *A_white_*. As a result, we obtained the following formulas for noise amplitude prediction:

(4a) $$A_{1/f}=-0.20FS-0.39R_{0}-0.59V_{b}+0.35{\left(FS\right)}^{2}-1.98,$$

(4b) $$A_{white}=-0.70FS+1.13R_{0}-0.86V_{b}-0.37R_{0}^{2}+0.26{\left(FS\right)}^{2}+0.22R_{0}\cdot FS-1.97,$$

where all variables are logarithmized using logarithm with base 10. An illustration of the formulas can be seen in Figure 5, where the prediction based on Equation (4a) and (4b) (red line) is plotted together with experimental data (black squares) for both 1/f-type noise (Figure 5a) and white noise (Figure 5b). The statistical coefficients of determination R^2^ were equal to approximately 0.86 for 1/f-type noise and 0.95 for white noise, which means that in both cases more than 85% of the experimental data variance can be explained using our model. The remaining part, which the model is unable to explain, can be attributed to measurement errors, insufficient precision or more complicated physical effects that cannot be captured by a simple regression approach.

The obtained noise levels can be used to determine quantities that are easily measurable in an experiment, for example the root mean square (RMS) voltage of noise *V_RMS_*. To obtain the expression for RMS in our model, Equation (1) can be rewritten in the following way:

(5) $$R\left(\theta \right)=R_{0}-∆R\cdot \cos\theta =R_{0}-∆R\cdot cos\left(\theta_{0}+A_{1/f}\varepsilon_{1/f}+A_{white}\varepsilon_{white}\right),$$

For small angle fluctuations and $\theta_{0}$ close to 90° (which is typical for highest sensitivity point on the transfer curve), we obtain:

(6) $$V\left(\varphi \right)\approx I_{b}\left(R_{0}+∆R\cdot \left(A_{1/f}\varepsilon_{1/f}+A_{white}\varepsilon_{white}\right)\right),$$

(7) $$V_{RMS}\approx I_{b}∆R\sqrt{{\left(A_{1/f}\right)}^{2}+{\left(A_{white}\right)}^{2}}.$$

By combining the obtained results, we can propose a simple method to calculate approximate value of *V_RMS_* in an TMR sensor based only on easily measurable quantities:

(1). Gather information about *R*_0_ (Ω), Δ*R* (Ω), *FS* (V/T) and *I_b_* (mA) from the experimental data. Typically, the last two quantities will be available in an explicit form. The first two may be intertwined with other parameters and available either only as information about resistances of parallel and anti-parallel state (*R_P_* and *R_AP_*) or as information about minimal and maximal voltage levels on the *U*(*H*) curve (*U_min_* and *U_max_*, respectively). In that case, one can recall that in our model *R*_0_ = (*R_AP_* + *R_P_*)/2 = (*U_max_* + *U_min_*)/(2·*I_b_*) and Δ*R* = (*R_AP_* − *R_P_*)/2 = (*U_max_* − *U_min_*)/(2·*I_b_*).
(2). Use Equation (4a) and (4b) to calculate *A*_1/*f*_ as well as *A_white_* values. It is important to note that these equations are given in a form where all variables are logarithmized using logarithm with base 10.
(3). Normalize the calculated *A*_1/*f*_ and *A_white_* values, dividing them by the sensing layer volume Ω expressed in μm^3^ units.
(4). Use the obtained *A*_1/*f*_ and *A_white_* together with Equation (7) to calculate *V_RMS_*.

**Figure 6:**
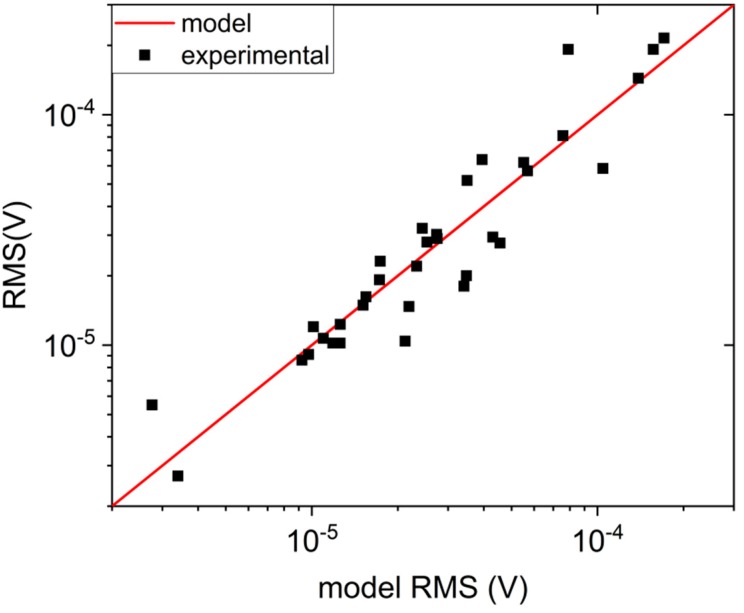
Root mean square value of voltage noise compared for model prediction (red line) and experimental data (black squares). The scale of both axes is logarithmic.

By following this procedure, we gathered model predictions for *V_RMS_* in all of our measured TMR sensor samples and compared them with the experimental data. The experimental *V_RMS_* values of noise were computed from power spectral noise density using the formula:

(8) $$V_{RMS,experimental}=\sqrt{\int_{f_{L}}^{f_{H}}S_{v}df},$$

where *f_L_* and *f_H_* are lowest and highest frequencies of measured noise, respectively, and *S_v_* is the noise power spectral density. We also computed standard deviation of the noise measured in time domain for a few selected sensors, and obtained *V_RMS_* values similar (difference below 10%) to the ones given by Equation (8). The comparison between experimental and model *V_RMS_* values can be seen below in the Figure 6. One can see that a good agreement is reached for nearly all measured samples, despite their very wide range of resistance and sensitivity values, which has a span of almost three orders of magnitude in both cases.

Overall, we would like to emphasize that the sensor data used to calibrate our model showed a great variety not only in terms of resistance and sensitivity, but also shape, size and sensing layer composition (see Table A2 in Appendix A). Therefore, we believe that our findings can be applied for most TMR sensors based on CoFeB/MgO/CoFeB structure with planar sizes on the order of 2–100 µm and typical sensing layer CoFeB compositions, regardless of their detailed shapes, composition ratios or sample preparation technologies.

## 4. Conclusions

We proposed a simple model for magnetic noise levels prediction in TMR sensors that is based on easily measurable quantities such as field sensitivity and resistance. Noise measurements for a wide variety of sensors, characterized by resistance values ranging from 13 Ω to 6250 Ω and field sensitivity values ranging from 0.3 V/T to 225 V/T, were performed. The experimental data were then used as the basis for a statistical approach based on polynomial regression together with cross-validation to identify the optimal formula for noise prediction. For both 1/f and white noise, we were able to explain over 85% of the experimental data variance using the obtained formula. We also calculated RMS value of noise and compared it with the values retrieved from the experimental data. The presented model can be used as a robust method for predicting approximate magnetic noise in TMR sensors based on basic experimental quantities.

## Appendix A

The overview of the gathered data can be seen in Table A1. Since the relevant experimental values differed from each other by multiple orders of magnitude, we decided to perform our analysis using logarithmized variables for resistance, sensitivity and current as well as for noise levels. Please note that the TMR ratios, which can be determined from the expression TMR = (*R_AP_* − *R_P_*)/*R_P_* = 2Δ*R*/(*R*_0_ − Δ*R*), are influenced by high bias voltages (up to 1000 mV). This happened because we were interested in devices working close to maximum sensitivity bias point, which typically requires higher voltage and thus decreased TMR value.

**Table A1:** Overview of measurement data and fitting results. Before logarithmizing, resistance values were given in Ohms, sensitivity values in V/T and current values in mA. The values of *A*_1/*f*_ and *A_white_* were normalized to 1 μm^3^ volume of the sensing layer.

No.	*R*_0_ (Ω)	Δ*R* (Ω)	*FS* (V/T)	*I_b_* (mA)	*Ω* (μm^3^)	*A*_1/*f*_	*A_white_*	log_10_ (*R*_0_)	log_10_ (Δ*R*)	log_10_ (*FS*)	log_10_ (*V_b_*)	log_10_ (*A*_1/*f*_)	log_10_ (*A_white_*)
1	6250	1250	1.9	0.008	0.029	8.02 × 10^−4^	1.79 × 10^−3^	3.80	3.10	0.28	1.70	−3.10	−2.75
2	6200	1200	3.8	0.017	0.029	6.17 × 10^−4^	9.87 × 10^−4^	3.79	3.08	0.58	2.02	−3.21	−3.01
3	5650	1150	22	0.09	0.041	1.36 × 10^−3^	9.26 × 10^−4^	3.75	3.06	1.34	2.71	−2.87	−3.03
4	5600	800	11	0.054	0.029	4.13 × 10^−4^	5.06 × 10^−4^	3.75	2.90	1.04	2.48	−3.38	−3.30
5	3950	825	1.65	0.0125	0.042	5.74 × 10^−4^	8.64 × 10^−4^	3.60	2.92	0.22	1.69	−3.24	−3.06
6	3800	400	25	0.21	0.029	4.50 × 10^−4^	6.05 × 10^−4^	3.58	2.60	1.40	2.90	−3.35	−3.22
7	3700	650	7.6	0.08	0.042	3.27 × 10^−4^	4.07 × 10^−4^	3.57	2.81	0.88	2.47	−3.49	−3.39
8	3400	500	10.4	0.15	0.042	3.33 × 10^−4^	8.64 × 10^−4^	3.53	2.70	1.02	2.71	−3.48	−3.06
9	1925	375	1.55	0.027	0.16	4.01 × 10^−4^	1.30 × 10^−3^	3.28	2.57	0.19	1.72	−3.40	−2.89
10	1925	375	1.52	0.027	0.16	3.46 × 10^−4^	9.26 × 10^−4^	3.28	2.57	0.18	1.72	−3.46	−3.03
11	1900	350	3.1	0.054	0.16	3.46 × 10^−4^	9.26 × 10^−4^	3.28	2.54	0.49	2.01	−3.46	−3.03
12	1900	350	2.83	0.055	0.16	2.53 × 10^−4^	8.02 × 10^−4^	3.28	2.54	0.45	2.02	−3.60	−3.10
13	1700	250	14.1	0.3	0.16	2.78 × 10^−4^	6.79 × 10^−4^	3.23	2.40	1.15	2.71	−3.56	−3.17
14	1700	250	9.2	0.32	0.16	1.48 × 10^−4^	3.02 × 10^−4^	3.23	2.40	0.96	2.74	−3.83	−3.52
15	1525	225	6	0.33	0.16	2.78 × 10^−4^	2.47 × 10^−4^	3.18	2.35	0.78	2.70	−3.56	−3.61
16	1150	125	23.6	0.89	0.16	1.48 × 10^−4^	5.31 × 10^−4^	3.06	2.10	1.37	3.01	−3.83	−3.28
17	1020	100	10.1	0.98	0.16	8.64 × 10^−5^	1.79 × 10^−4^	3.01	2.00	1.00	3.00	−4.06	−3.75
18	315	15	0.3	1.55	0.56	6.97 × 10^−5^	5.18 × 10^−4^	2.50	1.18	−0.52	2.69	−4.17	−3.29
19	110	25	127	4.5	0.92	1.30 × 10^−3^	1.60 × 10^−3^	2.04	1.40	2.10	2.69	−2.89	−2.79
20	107.5	22.5	110	4.8	0.92	2.72 × 10^−4^	1.36 × 10^−3^	2.03	1.35	2.04	2.71	−3.57	−2.87
21	105	20	58	4.6	0.92	1.54 × 10^−4^	9.26 × 10^−4^	2.02	1.30	1.76	2.68	−3.81	−3.03
22	102.5	22.5	120	5.2	0.92	1.05 × 10^−3^	1.17 × 10^−3^	2.01	1.35	2.08	2.73	−2.98	−2.93
23	100	15	12.7	5	0.92	7.40 × 10^−5^	4.13 × 10^−4^	2.00	1.18	1.10	2.70	−4.13	−3.38
24	100	15	6.5	5.1	0.92	2.47 × 10^−5^	3.09 × 10^−4^	2.00	1.18	0.81	2.71	−4.64	−3.51
25	100	15	2.7	4.9	0.92	3.09 × 10^−5^	1.85 × 10^−4^	2.00	1.18	0.43	2.69	−4.56	−3.73
26	90	25	23.3	0.55	3.61	1.48 × 10^−4^	7.40 × 10^−4^	1.95	1.40	1.37	1.69	−3.83	−3.13
27	72.5	12.5	158	6.6	3.61	4.75 × 10^−4^	3.76 × 10^−4^	1.86	1.10	2.20	2.68	−3.32	−3.42
28	50	9	6.8	9.9	2.50	3.09 × 10^−5^	1.11 × 10^−4^	1.70	0.95	0.83	2.69	−4.51	−3.95
29	48	10	23	10.2	2.50	4.94 × 10^−5^	2.10 × 10^−4^	1.68	1.00	1.36	2.69	−4.31	−3.68
30	48	8	3.9	10.4	2.50	1.85 × 10^−5^	1.05 × 10^−4^	1.68	0.90	0.59	2.70	−4.73	−3.98
31	46	7	2.3	10.2	2.50	1.23 × 10^−5^	1.23 × 10^−4^	1.66	0.85	0.36	2.67	−4.91	−3.91
32	17	3	1.54	30.5	9.90	4.94 × 10^−5^	3.70 × 10^−5^	1.23	0.48	0.19	2.71	−4.31	−4.43
33	15.5	1.5	0.32	32	9.31	1.23 × 10^−5^	5.55 × 10^−5^	1.19	0.18	−0.49	2.70	−4.91	−4.26
34	13	4	225	37	13.0	1.36 × 10^−4^	1.85 × 10^−5^	1.11	0.60	2.35	2.68	−3.87	−4.73

In addition to the measurement data, we present selected physical and technological characteristics of the sensors used for calibration in Table A2.

**Table A2:** Selected physical and technological characteristics of the TMR sensors used for calibration. In the sensing layer deposition method column, “standard” refers to DC standard magnetron sputtering and “linear dynamic” refers to linear dynamic DC magnetron sputtering deposition.

No.	Planar Shape	Planar Size (μm)	Sensing Layer Composition	Sensing Layer Deposition Method
1	rectangle	2.5 × 7.5	(Co_52_Fe_48_)_75_B_25_	standard
2	rectangle	2.5 × 7.5	(Co_52_Fe_48_)_75_B_25_	standard
3	square	5 × 5	Co_20_Fe_60_B_20_	linear dynamic
4	rectangle	2.5 × 7.5	(Co_52_Fe_48_)_75_B_25_	standard
5	rectangle	3 × 9	(Co_52_Fe_48_)_75_B_25_	standard
6	rectangle	2.5 × 7.5	(Co_52_Fe_48_)_75_B_25_	standard
7	rectangle	3 × 9	(Co_52_Fe_48_)_75_B_25_	standard
8	rectangle	3 × 9	(Co_52_Fe_48_)_75_B_25_	standard
9	rectangle	2 × 36	(Co_52_Fe_48_)_75_B_25_	standard
10	rectangle	2 × 36	(Co_52_Fe_48_)_75_B_25_	standard
11	rectangle	2 × 36	(Co_52_Fe_48_)_75_B_25_	standard
12	rectangle	2 × 36	(Co_52_Fe_48_)_75_B_25_	standard
13	rectangle	2 × 36	(Co_52_Fe_48_)_75_B_25_	standard
14	rectangle	2 × 36	(Co_52_Fe_48_)_75_B_25_	standard
15	square	10 × 10	Co_20_Fe_60_B_20_	linear dynamic
16	rectangle	2 × 36	(Co_52_Fe_48_)_75_B_25_	standard
17	rectangle	2 × 36	(Co_52_Fe_48_)_75_B_25_	standard
18	square	20 × 20	Co_20_Fe_60_B_20_	linear dynamic
19	circle	diameter 30	Co_60_Fe_20_B_20_	linear dynamic
20	circle	diameter 30	Co_60_Fe_20_B_20_	linear dynamic
21	circle	diameter 30	Co_60_Fe_20_B_20_	linear dynamic
22	circle	diameter 30	Co_60_Fe_20_B_20_	linear dynamic
23	circle	diameter 30	Co_60_Fe_20_B_20_	linear dynamic
24	circle	diameter 30	Co_60_Fe_20_B_20_	linear dynamic
25	circle	diameter 30	Co_60_Fe_20_B_20_	linear dynamic
26	circle	diameter 60	Co_60_Fe_20_B_20_	linear dynamic
27	circle	diameter 60	Co_60_Fe_20_B_20_	linear dynamic
28	circle	diameter 50	Co_60_Fe_20_B_20_	linear dynamic
29	circle	diameter 50	Co_60_Fe_20_B_20_	linear dynamic
30	circle	diameter 50	Co_60_Fe_20_B_20_	linear dynamic
31	circle	diameter 50	Co_60_Fe_20_B_20_	linear dynamic
32	square	90 × 90	Co_40_Fe_40_B_20_	linear dynamic
33	square	90 × 90	Co_40_Fe_40_B_20_	linear dynamic
34	square	100 × 100	Co_40_Fe_40_B_20_	linear dynamic
